# Interspecific Recombination Between Zucchini Tigre Mosaic Virus and Papaya Ringspot Virus Infecting Cucurbits in China

**DOI:** 10.3389/fmicb.2021.773992

**Published:** 2021-11-03

**Authors:** Bin Peng, Liming Liu, Huijie Wu, Baoshan Kang, Zhangjun Fei, Qinsheng Gu

**Affiliations:** ^1^Zhengzhou Fruit Research Institute, Chinese Academy of Agricultural Sciences (CAAS), Zhengzhou, China; ^2^Boyce Thompson Institute, Ithaca, NY, United States; ^3^United States Department of Agriculture-Agricultural Research Service, Robert W. Holley Center for Agriculture and Health, Ithaca, NY, United States

**Keywords:** interspecific recombinant, zucchini tigre mosaic virus, papaya ringspot virus, cucurbits, evolution

## Abstract

Recombination drives evolution of single-stranded RNA viruses and contributes to virus adaptation to new hosts and environmental conditions. Intraspecific recombinants are common in potyviruses, the largest family of single-stranded RNA viruses, whereas interspecific recombinants are rare. Here, we report an interspecific recombination event between papaya ringspot potyvirus (PRSV) and zucchini tigre mosaic potyvirus (ZTMV), two potyviruses infecting cucurbit crops and sharing similar biological characteristics and close phylogenetic relationship. The PRSV-ZTMV recombinants were detected through small RNA sequencing of viruses infecting cucurbit samples from Guangxi and Henan provinces of China. The complete nucleotide (nt) sequences of the interspecific recombinant viruses were determined using overlapping RT-PCR. Multiple sequence alignment, recombination detection analysis and phylogenetic analysis confirmed the interspecific recombination event, and revealed an additional intraspecific recombination event among ZTMV populations in China. The symptoms and host ranges of two interspecific recombinant isolates, KF8 and CX1, were determined through experimental characterization using cDNA infectious clones. Surveys in 2017 and 2018 indicated that the incidences of the interspecific recombinant virus were 16 and 19.4%, respectively, in cucurbits in Kaifeng of Henan province. The identified interspecific recombinant virus between PRSV and ZTMV and the novel recombination pattern with the recombination site in HC-pro in *potyvirid* provide insights into the prevalence and evolution of ZTMV and PRSV in cucurbits.

## Introduction

Cucurbits including watermelon (*Citrullus lanatus*), melon (*Cucumis melo*), cucumber (*Cucumis sativus*), zucchini (*Cucurbita pepo*), and pumpkin (*Cucurbita moschata*) are economically important fruit and vegetable crops worldwide. According to FAO statistics in 2018, China was the world’s largest producer and consumer of major cucurbit crop products, exceeding 60% of production and consumption globally. Virus diseases pose a major threat to cucurbit crop production, and more than 90 species of viruses have been reported to infect cucurbits worldwide (Liu et al., 2019b). So far, 15 potyviruses, one of the largest groups of plant viruses, have been reported to naturally infect cucurbits: Algerian watermelon mosaic virus (AWMV), clover yellow vein virus (ClYVV), cucurbit vein banding virus (CVBV), melon vein-banding mosaic virus (MVBMV), Moroccan watermelon mosaic virus (MWMV), papaya ringspot virus (PRSV), Sudan watermelon mosaic virus (SuWMV), turnip mosaic virus (TuMV), watermelon leaf mottle virus (WLMV), watermelon mosaic virus (WMV), wild melon vein banding virus (WMVBV), zucchini yellow fleck virus (ZYFV), zucchini yellow mosaic virus (ZYMV), zucchini tigré mosaic virus (ZTMV), and zucchini shoestring virus (ZSSV; Lecoq and Desbiez, 2012; Romay et al., 2014; Ibaba et al., 2016; Perotto et al., 2018; Moury and Desbiez, 2020). More than 30 viruses infect cucurbit crops in China, leading to a loss of nearly 4.2 billion dollars each year (Liu et al., 2019b).

Zucchini tigre mosaic potyvirus is a member of the genus *Potyvirus*, containing genomic RNA of ~10.3kb in length, which encodes two ORFs, the pretty interesting potyvirus open reading frame (ORF; encoding protein PIPO) and a single major polyprotein that is cleaved into 10 mature proteins: protein 1 (P1), helper component-protease (HC-Pro), protein 3 (P3), 6-kDa peptide 1 (6K1), Cylindrical Inclusion protein (CI), 6-kDa peptide 2 (6K2), Viral Protein genome-linked (VPg), Nuclear inclusion A-protease (Nla-pro), Nuclear inclusion B (Nlb), and Coat protein (King et al., 2011; Romay et al., 2014). The PRSV cluster, first defined in 2008 (Yakoubi et al., 2008), contains a group of cucurbit-infecting potyviruses closely related to PRSV biologically, serologically and molecularly. So far, the cluster contains eight documented viruses: AWMV, MWMV, PRSV, SuWMV, WMVBV, ZSSV, ZTMV, and ZYFV (Desbiez et al., 2017). ZTMV, first described in Guadeloupe Island in the Caribbean region, is designated as the PRSV type T due to the differences from PRSV in serology and its symptoms of discoloration resembling a tiger stripe pattern on leaves of zucchini (Romay et al., 2014). According to phylogenetic analysis and sequence comparison, PRSV is the most closely related potyvirus with ZTMV, and their complete genomes share 66.8–68% nt sequence identities. Therefore, based on molecular and biological evidence, ZTMV has been proposed as a distinct potyvirus in the PRSV cluster (Quiot-Douine et al., 1986; Romay et al., 2014). ZTMV has been reported in three continents (Asia, Europe, and America) and in some Caribbean and Indian Ocean islands (Romay et al., 2014; Xiao et al., 2016; Desbiez et al., 2017; Abdalla and Ali, 2018; Wang et al., 2019). Based on phylogenetic analysis of the complete CP nt sequence, ZTMV isolates are classified into three subgroups: Asian, Indian Ocean, and American, related to their geographical origins (Romay et al., 2014). In 2014, the first Chinese isolate of ZTMV was reported in cucurbit plants from Yunnan province of China, which was identified as a major pathogen affecting cucurbit production in Yunnan province due to its wide distribution (Xiao et al., 2016). Moreover, two ZTMV isolates infecting wax gourd (*Benincasa hispida*) under accession number MN267689 and bitter melon (*Momordica charantia*) under accession number LC371337 have been detected in Guangdong and Taiwan provinces, respectively.

Numerous recombination events are involved in potyvirus evolution (Valli et al., 2007; Salvador et al., 2008). However, in a recombination analysis of 152 ORF sequences encoding polyprotein of potyvirus, only five interspecific recombination events were found, of which only two, WMV and SuWMV, were confirmed with experimental evidence (Gibbs et al., 2020). WMV is a recombinant between soybean mosaic virus (SMV) and bean common mosaic virus (BCMV; Desbiez and Lecoq, 2004), and SuWMV originated from an interspecific recombination event between a WMVBV and a MWMV (Desbiez et al., 2017). The junctions of these two recombination events are both in the coding region of P1, the first protein encoded by the potyvirus genome. P1 functions in biological processes such as virus replication, cell-to-cell movement, and systemic spread and stimulation of the gene silencing suppressor HC-Pro (Rohožková and Navrátil, 2011). In addition, previous studies have suggested that recombination site in the N-terminal of the P1 coding region of potyvirus could be less deleterious than that in other regions of the viral genomes, and therefore facilitate its adaptation to a wide range of host species (Valli et al., 2007; Salvador et al., 2008; Pasin et al., 2014; Desbiez et al., 2017).

In this study, three recombinant cucurbit-infecting ZTMV isolates identified in China are described and their biological properties were also investigated using infectious cDNA clones.

## Materials and Methods

### Sample Collection

Sixty cucurbit leaf samples were collected in July 2017 and 2018 from Xinhuaying Town, Kaifeng City, Henan Province (35°29'31''N, 114°45'39''E) and in September 2017 from Anping Town, Cenxi County, Guangxi Zhuang Autonomous Region (23°7'10''N, 111°4'35''E). All samples, including six melon, 21 pumpkin and 33 watermelon leaf samples, exhibited virus-like symptoms such as mosaic, mottle, yellows, or crinkle (Supplementary Table 1). The collected samples preserved in dry ice were shipped to the lab and then stored at −80°C.

### Small RNA Sequencing

Total RNA was extracted from frozen leaves using the RNAiso Plus kit (Takara, China) following the manufacturer’s instructions. The quantity and integrity of total RNA were determined using a Qubit2.0 analyzer (Bioptic, China) and an Agilent 2100 Bioanalyzer (Agilent Technologies, United States), respectively. Seven small RNA (sRNA) mixtures (SR1-SR7), each pooled from 2 or 3 samples out of the 16 selected cucurbit samples according to the collected fields, hosts, and symptoms (Supplementary Table 2), were subjected to sRNA sequencing. Small RNA preparation and library construction, using the NEBNext® Small RNA Library Prep Set for Illumina® (NEB, United Kingdom), were performed following the manufacturer’s instructions. Briefly, unique barcoded adaptors were added, RT-PCR was performed and the product was purified *via* polyacrylamide gel electrophoresis. sRNA libraries were sequenced on an Illumina NextSeq 500 system.

### sRNA Read Processing and Analysis for Virus Identification

The adaptors of raw sRNA reads were removed using the perl script included in the VirusDetect package (Zheng et al., 2017). Cleaned sRNA reads, excluding short (<15nt) and low-quality reads (<Q30), were subjected to virus detection using VirusDetect (V1.70), which performs reference-guided assembly by aligning sRNA reads to the known virus reference database (GenBank gbvrl) as well as *de novo* assembly using Velvet (Zerbino and Birney, 2008) with automated parameter optimization. Virus database v229 (included in VirusDetect package) was used as the virus reference sequences for the reference-guided assembly. Melon (Garcia-Mas et al., 2012), watermelon (Guo et al., 2013) and *Cucurbita moschata* (Sun et al., 2017) genomes were used to subtract sRNA reads derived from the cucurbit hosts.

### Complete Genome Sequencing

**Figure 1:**
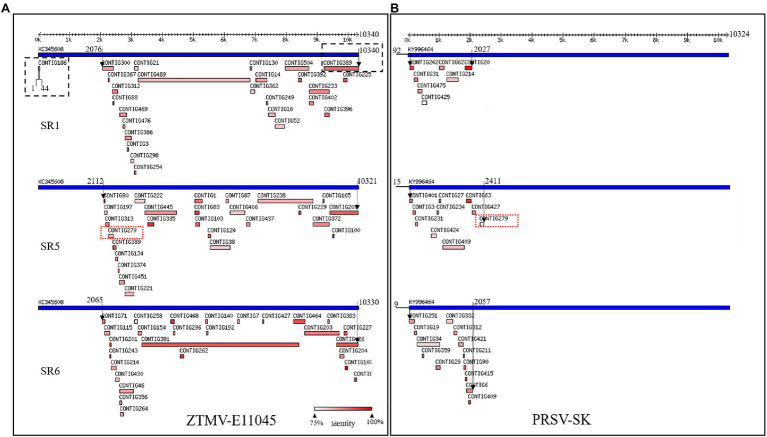
Viruses identified from SR1, SR5, and SR6 samples using VirusDetect. **(A)** Alignments of assembled virus contigs to the ZTMV-E11045 genome. **(B)** Alignments of assembled virus contigs to the PRSV-SK genome. Blue tracks represent reference virus genomes, and red tracks represent assembled virus contigs. Double headed arrows with scales represent the length of genome. Black arrows represent range of contigs aligned with reference genome. Black dotted line box represents contigs aligned to the terminals of zucchini tigre mosaic potyvirus (ZTMV) reference genome. Red dotted line boxes represent contig279 from SR5 aligned to both ZTMV and PRSV reference genomes.

**Figure 2:**
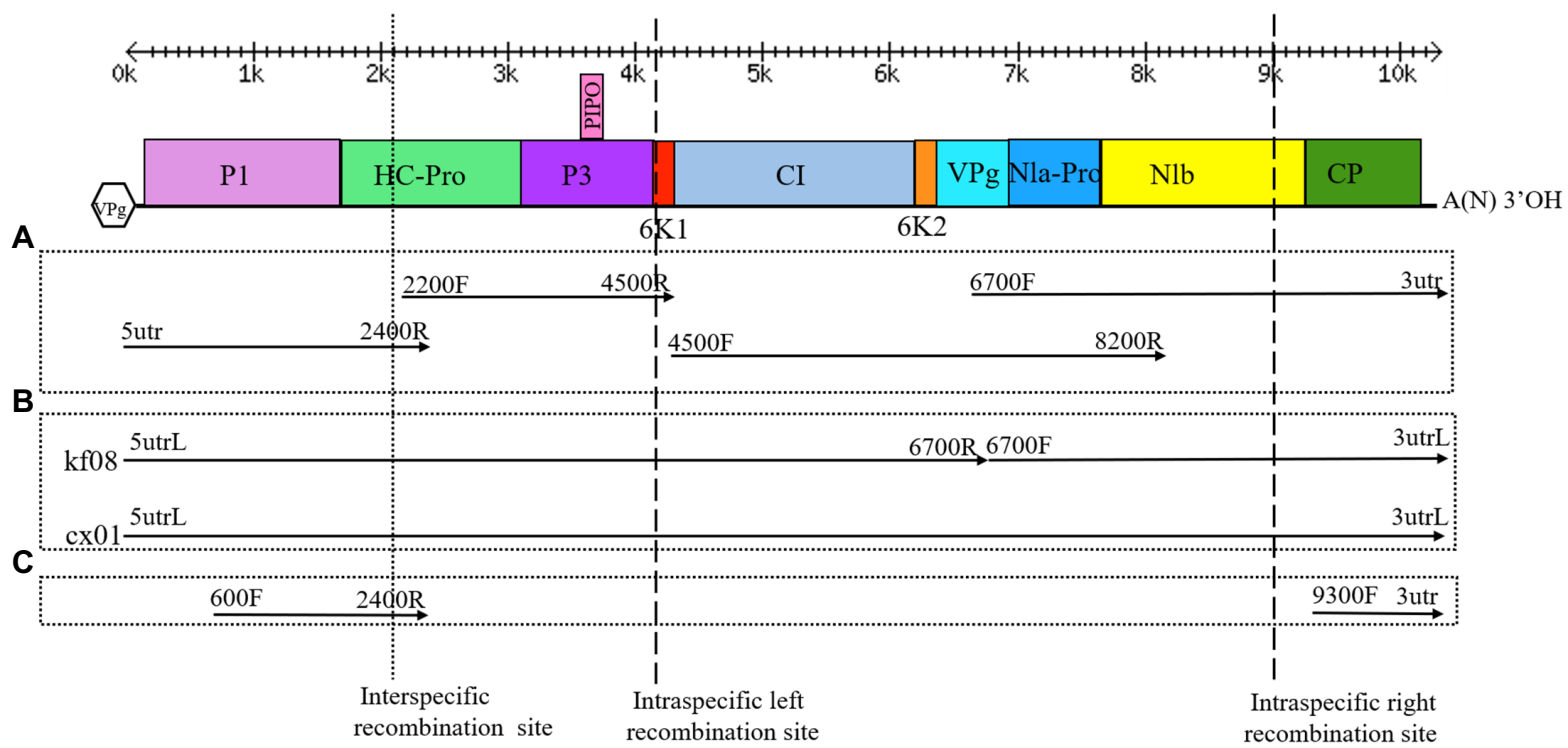
Genome organization of ZTMV, detection of recombination isolates, cloning of complete genomes and construction of infectious cDNA clones. **(A)** Strategy of cloning the complete genomes. **(B)** Strategy of detection of recombination and construction of infectious cDNA clone. **(C)** Strategy of detection of recombination isolate of ZTMV from samples in field. Double-headed arrows with scales represent the length of the genome. Blackline represents the genome. Colored rectangles represent matured proteins derived from the large polyproteins. The Vpg in hexagon represents viral protein genome-linked. A(N)3'OH represents the polyA tail. Arrows represent the PCR fragments that were cloned. Primers used for amplifying the PCR fragments are indicated at the ends of each arrow. The cross of dotted line and the genome indicates the interspecific recombination point. The crosses of dash lines and the genome indicate the intraspecific recombination points.

According to the mapping of virus contigs assembled by VirusDetect to the reference genomes of ZTMV, a series of primers were designed to obtain the full genome sequence of ZTMV (Supplementary Table 3). RACE (rapid amplification of cDNA ends) was performed to determine the missing 5' and 3' end sequences of isolates using a SMARTer® RACE 5'/3' Kit (Clontech, United States) according to the manufacturer’s instructions. The primer ZTMV-3utr, complementary to the 3' terminus of the genome of ZTMV, was used to generate cDNA by GoScript reverse transcriptase (Promega, United States). The 5' termini of KF17 and CX1 were determined by RACE using net primer sets 600R and 800R, respectively. The 5' and 3' terminus nt sequences of KF8 were successfully assembled from sRNA reads using VirusDetect (Figure 1A). The complete nt sequences of the three isolates (KF8, KF17, and CX1) of ZTMV were confirmed by Sanger sequencing of four segments derived from four overlapping RT-PCRs using four primer pairs (5utr&2400R, 2200F&4500R, 4500F&6700R, and 6700F&3utr shown in Figure 2A). Complete nt sequences of KF8 and CX1 isolates were further confirmed by one or two long-distance PCRs (LD-PCRs; primers pair shown in Figure 2B) and Sanger sequencing. Conventional PCRs, whose products were less than 3kb, were performed using the 2×Phanta Master Mix (Vazyme, China), and LD-PCRs, whose products were more than 3kb, were done using the 2×Phanta Max Master Mix (Vazyme, China). PCR products were cloned using the CV17-Zero Background pTOPO-Blunt Simple Cloning Kit (Aidlab, China) or into the pXT1 vector (Yao et al., 2011), and transformed into competent cells *Escherichia coli* Top10. Three independent clones or PCR products were selected for Sanger sequencing (Sangon, China). The assembly of the complete genome sequence was performed using the sequence assembly tools in the DNAMAN package (Version 8).^1^ The prediction of ORF and translation of polyproteins were performed using the ORFfinder.^2^ Identities of nucleotide sequences of the viral genome and UTR, as well as amino acid sequences of Polyprotein and 10 mature proteins, were derived from the multiple sequence alignments by Clustal Omega (Sievers and Higgins, 2014).

### Recombination Analysis

**Figure 3:**
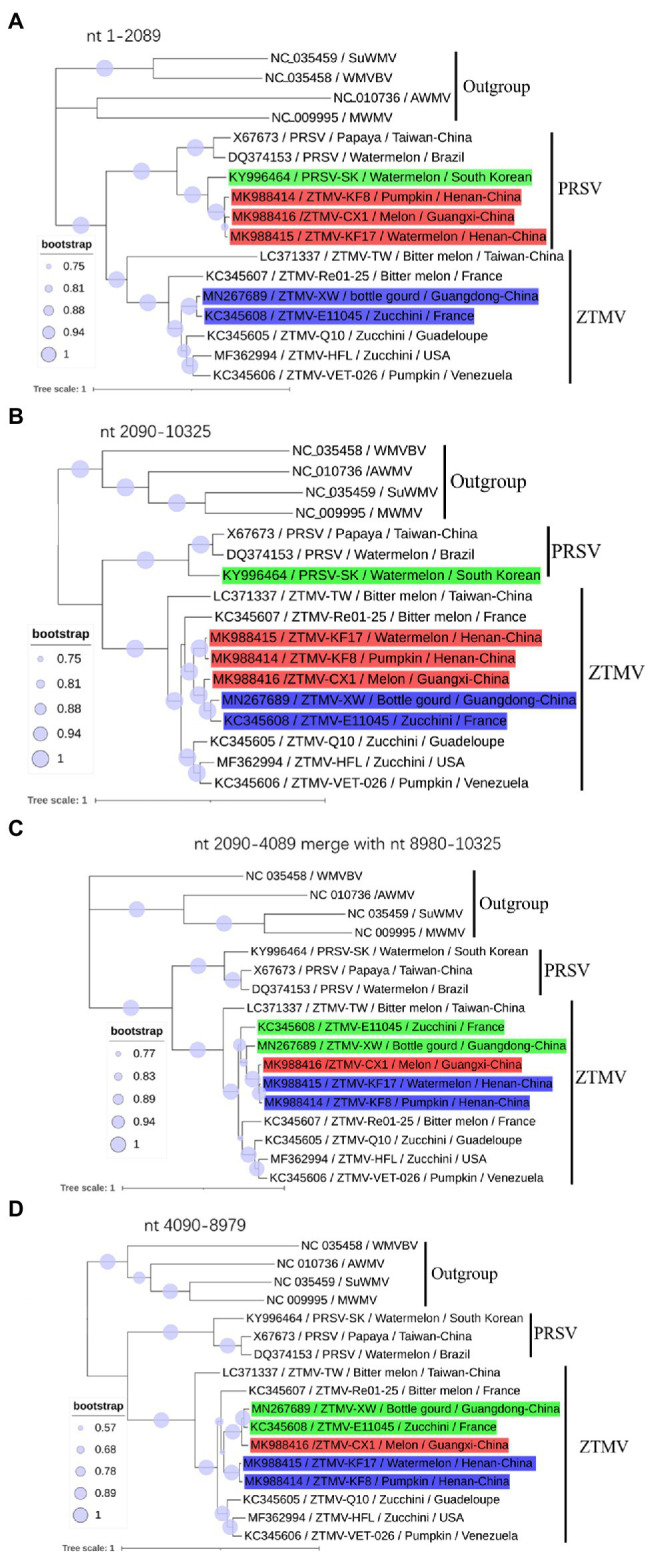
Phylogenetic analysis of non-recombinant regions. **(A)** Phylogenetic tree based on nt 1–2,089 sequences; **(B)** Phylogenetic tree based on nt 2,090–10,325 sequences; **(C)** Phylogenetic tree based on nt 2,090–4,089 merged with nt 8,980–10,325 sequences. **(D)** Phylogenetic tree based on nt 4,089–8,079. Accession number, isolate name, host, and collected location are provided in nodes. Isolates with red, green, and blue backgrounds represent recombinant isolates, minor parents and major parents in the interspecific recombinant event, respectively.

Analyses of recombination of 13 viral genome sequences, including all 10 ZTMV available in GenBank and three PRSV complete sequences (Accession numbers and isolates names listed in Figure 3), were performed using RDP (version 4.8), which includes seven recombinant detection algorithms: RDP, GENECONV, BoosScan, MaxChi, Chimaera, SiScan, and 3Seq (Martin et al., 2015). We considered only the potential recombinant events with an average value of *p* <0.01 in four or more recombinant detection algorithms.

### Phylogenetic Analysis

According to the recombination pattern derived from the RDP analyses, the recombinant events were confirmed by phylogenetic analysis. Prior to the phylogenetic tree construction, jModelTest2 (Darriba et al., 2012) was used to select the best fitting evolution model for the alignment. Phylogenetic trees were constructed using MEGA X (Kumar et al., 2018) with the maximum-likelihood method and 500 bootstrap replicates, and visualized in iTOL.^3^ All full-length sequences of ZTMV isolates retrieved from GenBank and determined in this study were included in the phylogenetic analysis. Meanwhile, three representative PRSV isolates and four *potyviruses* (AWMV, MWMV, SuWMV, and WMVBV) regarded as closely related to PRSV were used as the outgroup. The same phylogenetic analyses were applied to nt sequences of coding region of CP and CI, respectively.

### Construction of Infectious cDNA Clones

Since the plant samples infected with ZTMV were also infected by other viruses including ZYMV and WMV, the isolation of ZTMV from these samples was performed with the construction of infectious cDNA clones. The samples 2017-kf08 and 2017-cx01, collected from Henan province and Guangxi Zhuang Autonomous Region, respectively, were propagated in zucchini (*Cucurbita pepo*) cv. Green Beauty plants. The extraction of RNA and synthesis of first cDNA were as described above. To construct infectious cDNA clones of ZTMV from sample 2017-kf08, two fragments of 6.7 and 3.6kb in length were amplified using 5utrL&6739R and 6700F&3utrL primers pairs, respectively (Supplementary Table 3) by LD-PCR described above. For 2017-cx01, one fragment covering the genome sequence was amplified by LD-PCR using the 5utrL&3utrL. The two fragments from 2017-kf08 and one fragment from sample 2017-cx01 were assembled with the *Stul*I and *Sma*I-treated pXT1 vector (Yao et al., 2011) using ClonExpress MultiS one step cloning kit (Vazyme, China). The *Agrobacterium*-mediated inoculation method for cucurbit plants was as described in Liu et al. (2019a). Briefly, plasmids were introduced into *Agrobacterium tumefaciens* freeze-thawed with liquid nitrogen. After bacterial growth and induction, cell suspension of agrobacterium was injected into cotyledons of muskmelon. Plants injected with *Agrobacterium* containing empty vector plasmid pXT1 were used as the negative control.

### Validation of Infectivity

Each of the two infectious cDNA clones and the empty vector was inoculated on 10 melon plants. Inoculated plants were monitored until the occurrence of apparent symptoms or till 30days post-inoculation. To confirm the presence of ZTMV in plants, two RT-PCR detections were carried out using primer pairs of 4500F&6400R and 8300F&3utr, respectively. Meanwhile, the RNA of ZTMV was detected with Northern dot-blot analysis using the DIG Northern Starter Kit (Roche, German). To synthesize the probe for dot-plot analysis, a RT-PCR product was obtained using the RNA from kf08 sample as the template and primers 9292F&T7_10064R, and then transcribed into RNA, which was labeled with DIG in an *in vitro* transcription reaction following the kit manual. For dot-plot analysis, in brief, total RNA extracted from 0.1g fresh leaves of test plants was fixed in Hybond-N membranes at 120°C for 30min. The fixed membranes were then hybridized with a DIG-labeled RNA probe and photographed using the Chemiluminescence imaging system (Tanon, China). The RNA from leaves infected by ZTMV in fields and non-inoculated leaves of plants injected with Agrobacterium with empty vector plasmid pXT1 were used as the positive and negative controls, respectively. Screening of other viruses infecting cucurbits, including ZYMV, WMV, PRSV, and CMV, was carried out with species-specific primers and commercial double-antibody sandwich enzyme-linked immunosorbent assay (DAS-ELISA) kit (Agdia, United States), to make sure that the tested cucurbit plants were free of these viruses. Conventional RT-PCR was performed as described above. DAS-ELISA was performed following the instructions, and the criterion used to demarcate the positive or negative results was determined according to Liu et al. (2017).

### Validation of Aphid Transmissibility

One gram fresh leaves from melon plant inoculated by infectious cDNA clones were ground with 10ml of 0.01M PBS (pH 7.0). The prepared inocula were mechanically inoculated on cotyledons of 15 melon plants. The timing and symptoms were recorded after 5days post inoculation (dpi) till the appearance of viral-disease symptoms. In March 2018, melon aphids, *Aphis gossypii* Glover (Hemiptera: Aphididae), were collected from symptomless melon, grown in a tunnel located in Zhengzhou, Henan province. After transfer to melon plants grown in air-conditioned insect-proof glasshouse with 18–24°C, 20 apterous aphids were maintained and propagated. Prior to aphid transmission tests, the aphids were validated as virus-free by DAS-ELISA and RT-PCR using commercial ELISA kit and species-specific primers for ZYMV, WMV, CMV, and PRSV. Before being transferred to symptomatic and systemically ZTMV-infected melon plants, the aphids were gently transferred into a petri dish by brush pen and starved for 30min. The aphids were fed on ZTMV-infected melon for 5–10min acquisition access periods followed by transfer to healthy melon plants (10–15 aphids per plant) for inoculation access periods of 24h. Sulfoxaflor, a quick-acting insecticide against aphid, was applied to kill them. Fifteen melon plants were aphid-inoculated with two ZTMV isolates. Virus-free aphids, fed on healthy melon plants, were transferred to another 10 melon plants as the healthy controls. The symptoms that developed were recorded. To detect the presence of ZTMV, the top young leaf was taken from each plant at 14 dpi and RT-PCR was carried out using ZTMV species-specific primers as described above.

### Host Range Determination

Mechanical inoculation of 12 plant species, including seven *Cucurbitaceae* crops, five indicative plants and papaya (Table 1), was used to determine an experimental host range and symptoms for two isolates of ZTMV. The inocula were prepared and inoculated on cotyledons of cucurbit plants as described above. The top three fully expanded leaves of the five indicative plants (at the 6-leaf stage) and papaya (at the 8-leaf stage) were inoculated with the same inocula. At least10 plants of each species were used in the experiment. For inoculated plants at 30 dpi that showed no symptom, the absence of the virus was confirmed by RT-PCR. Plant inoculations were carried out with three independent repeats.

**Table 1:** Host ranges of ZTMV-KF8 and ZTMV-CX1 and symptoms of 13 plant species.

Host	Cultivar	ZTMV-KF8		ZTMV-CX1	
		Positive/total	Symptoms	Positive/total	Symptoms
*Cucurbita pepo*	Green beauty	10/10	M, Ma	10/10	M, Ma
*Cucurbita moschata*	Miben	6/10	B, Ma	7/10	B, Ma
*Citrullus lanatus*	Zhengkang No.2	8/10	M, Mo	5/10	M, Mo
*Cucumis melo Makuwa*	Qingtian	7/10	YS, Cu	10/10	YS, Cu
*Cucumis melo*	Queen	10/10	M	10/10	M
*Cucumis sativus*	Dongyu	6/10	MM	10/10	MM
*Lagenaria siceraria*	Yongzhen No.1	8/10	M, Ma	5/10	M
*Nicotiana benthamiana*	–	0/30	ns	0/30	ns
*Nicotiana glutinosa*	–	0/30	ns	0/30	ns
*Nicotiana occidentalis*	–	0/30	ns	0/30	ns
*Chenopodium Amaranticolor*	–	0/30	ns	0/30	ns
*Chenopodium quinoa*	–	0/30	ns	0/30	ns
*Carica papaya*	FZ No.1	0/30	ns	0/30	ns

M, mosaic; MM, mild mosaic; Mo, mottle; YS, yellow spot; Ma, malformation; B, blistering; Cu, curl; ns, no symptom. “positive” indicates the number of positive plants detected by RT-PCR; “total” indicates the number of total tested plants.

### Detection of Recombinants by RT-PCR From Samples in the Field

Conventional RT-PCR was used for detection of ZTMV from 60 samples, collected in 2017 and 2018 (Supplementary Table 1), using two primer pairs, 600F&2400R that covered the interspecies recombination site, and 8300F&3utr (Supplementary Table 3), targeting partial P1-HC-pro region and CP coding region, respectively.

## Results

### Small RNA Sequencing

After removing adapters, short and low-quality reads, we obtained 14,874,695–23,160,143 cleaned reads for each of the seven RNA mixture (SR1–SR7; Supplementary Table 2). Using these sRNA data, nine viruses were identified with the VirusDetect pipeline (Zheng et al., 2017), including ZYMV, WMV, ZTMV, PRSV, melon aphid-borne yellows virus (MABYV), *Cucumis melo* alphaendornavirus (CMEV), squash leaf curl china virus (SLCCV), melon yellow spot virus (MYSV), and *Citrullus lanatus* cryptic virus (CiLCV). Except for ZTMV and PRSV, the coverage of assembled contigs of all viruses against the corresponding reference genomes was greater than 95% (Supplementary Table 2). For the sRNA data of SR1, SR5, and SR6, 26, 26, and 27 contigs were aligned to the genome sequence of ZTMV E11045 isolate (Accession number KC345608), respectively, covering 78.6, 68.3, and 79.1% of the E11045 genome with an average sequence identity of 87.7, 88.7, and 92.2% (Supplementary Table 4). Except for one contig from SR1, which was aligned against nt 1–44 of the ZTMV-E11045 sequence, all contigs covered the range from about nt 2,000 to the 3' end of the genome. There was a about 2,000-nt gap from the 5' end to 2,000nt where no contigs were aligned to the reference ZTMV genome (Figure 1A). Furthermore, 7, 10, and 13 contigs from SR1, SR5, and SR6, respectively, were aligned to the genome sequence of isolate PRSV-SK (Accession number KY996464). They covered 11.9, 16.7, and 17.6% of the PRSV-SK genome with sequence identities of 85.4, 87.1, and 88.5%, respectively. All these contigs only covered the 5' end to about 2,000nt in the reference sequence (Figure 1B). Interestingly, contig279 from SR5 was not only aligned to PRSV with 80% identity but also to ZTMV with 85% identity.

### Complete Sequencing and Sequence Alignment of KF8, KF17, and CX1

**Figure 4:**
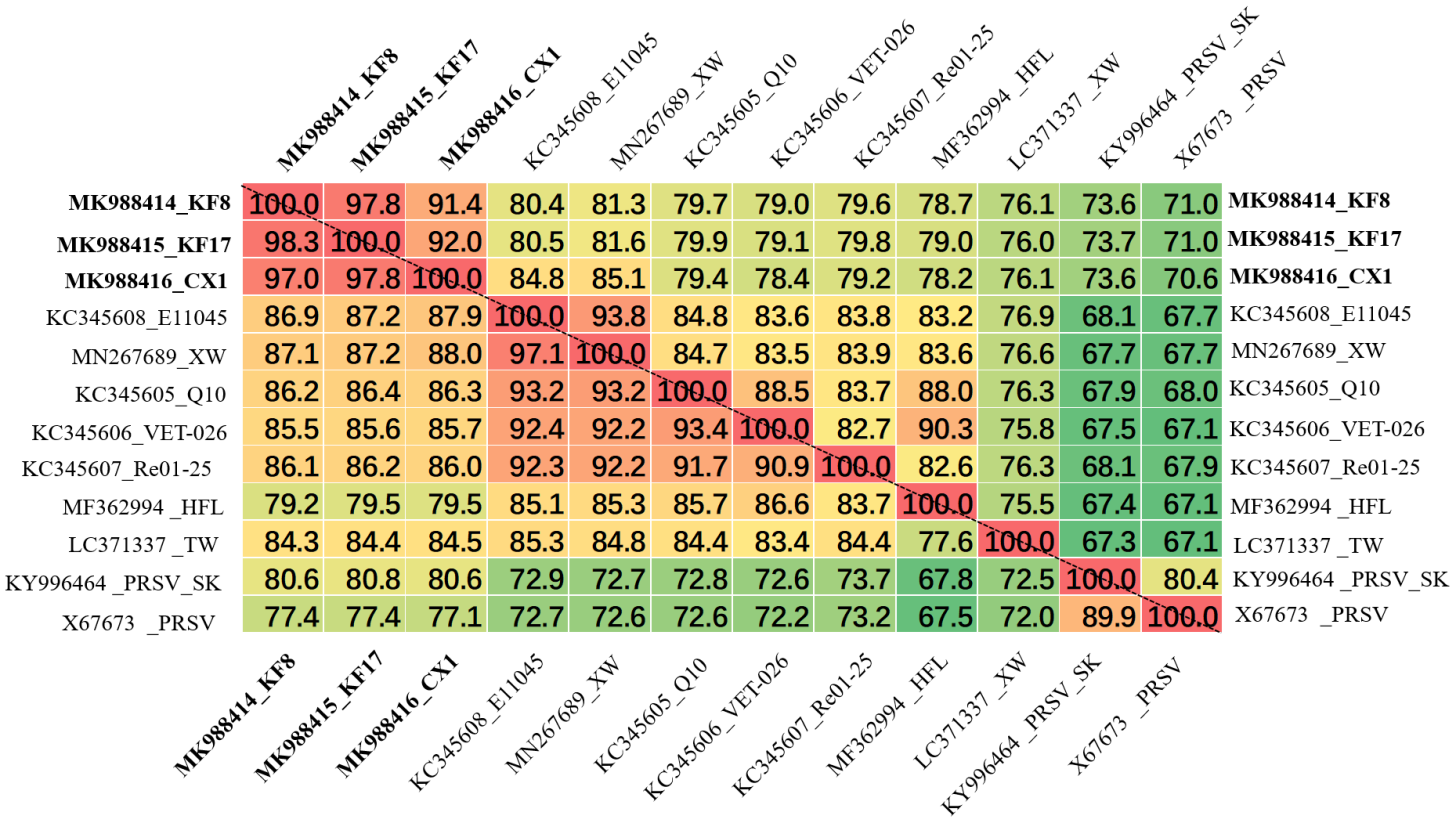
Matrix of percent identity between the complete genome sequence (above the dotted-line) and polyprotein (below the dotted-line) of three recombination isolates and other ZTMV and two PRSV isolates.

The ZTMV isolates from samples 2017-kf08, 2017-kf17, and 2017-cx01 were named as KF8, KF17, and CX1, respectively. The complete nt sequences of ZTMV-KF8, ZTMV-KF17, and ZTMV-CX1 were found to be 10,325, 10,328, and 10,331 nucleotides in length, respectively, excluding the poly-(A) tail (Supplementary Figures 1B,C). They all presented a typical potyvirus genome organization, including a 5'-UTR, a single large ORF encoding a polyprotein, a motif for the PIPO protein and a 3'-UTR (Figure 2). The complete nucleotide sequences of KF8, KF17, and CX1 have been deposited in GenBank under accession numbers MK988414, MK988415, and MK988416, respectively. There were some variations in length of the genome, 5'-UTR, polyprotein and 3'-UTR comparing the three isolates with other ZTMV and two PRSV isolates from GenBank (Supplementary Table 5). The complete nt and aa sequences of KF8, KF17, and CX1 shared 91.4–97.8% and 97–98.3% identities between each other, 76.1–81.3% and 85.2–87.1% identities with other isolates of ZTMV from GenBank (excluding the HFL isolate from Hawaii), and less than 73.6 and 80.6% identities with the two isolates of PRSV (Figure 4). These results suggest that the three isolates, KF8, KF17, and CX1, should belong to ZTMV, according to the species demarcation criteria of *Potyviridae* (Wylie et al., 2017). The alignment information of nucleotide sequences of 5'- and 3'-UTRs and amino acid sequences of 10 mature proteins (P1, HC-pro, P3, 6K1, CI, 6K2, Vpg, Nla-pro, Nlb, and CP) of KF8 against those of KF17, CX1, other ZTMV isolates and two PRSV isolates is provided in Supplementary Table 6. There were two unexpected results in the 5'-UTR region and P1 protein. The 5'-UTR sequences of the KF8 isolate were both 97.6% identical to that of KF17 and CX1, and 52.4–65.9% identical to that of other seven ZTMV from GenBank, but 86.6 and 67.1% identical to that of PRSV-SK and the reference sequence of PRSV. Similarly, the amino acid sequence of P1 of the KF8 isolate was 94.2 and 92.3% identical to that of KF17 and CX1, 37.3–40.3% identical to that of other seven ZTMV from GenBank, while 83.4 and 64.4% identical to that of PRSV-SK and the reference sequence of PRSV (Supplementary Table 6). These results revealed that the three isolates could be recombinant isolates.

### Recombination Analyses

**Figure 5:**
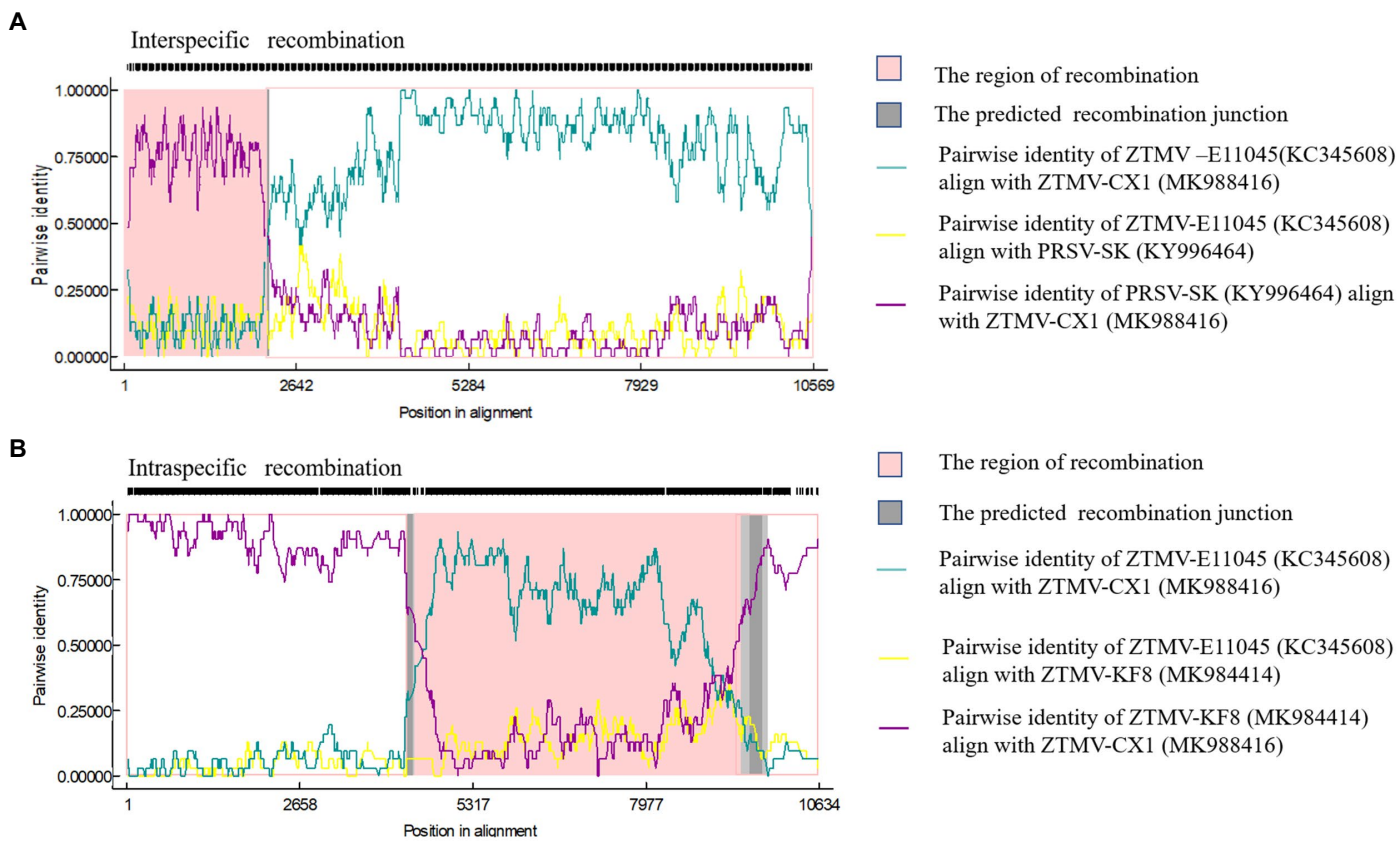
Recombination in the ZTMV genome predicted by RDP4. **(A)** Nucleotide sequence identity along the genome of ZTMV-CX1, ZTMV-E11045, and PRSV-SK. **(B)** Nucleotide sequence identity along the genome of ZTMV-CX1, ZTMV-E11045, and ZTMV-KF8.

Complete sequences of 10 ZTMV and three PRSV isolates were subjected to recombination analysis using RDP4 (Martin et al., 2015). Two strong independent recombination signals (value of *p* <10^−11^; Table 2), one interspecific (Figure 5A) and one intraspecific (Figure 5B), were identified by all seven methods used in RDP4. The three isolates, KF8, KF17, and CX1 from China, were regarded as interspecific recombination products, derived from a virus related of SK isolate of PRSV (Accession number KY996464) from south Korean and a virus related of ZTMV isolates E11045 from France (Accession number KC345608) or XW from Guangdong of China (Accession number MN267689; Figure 5A). The interspecific recombination junction was located in the genome of CX1 isolate at around position nt 2,089 within the HC-pro coding region (Figure 2). The front region of its complete sequence, including 5'-UTR, P1 and part of HC-Pro, was derived from PRSV, and other regions were derived from ZTMV. Another intraspecific recombination isolate, CX1, was derived from an intraspecific event (Figure 5B). The junctions were located in the CX1 genome at around nt 4,089 within the 6K1 coding region and nt 8,980 within the Nlb coding region (Figure 2). Full-length amplification using LD-PCR (Figure 2B) and Sanger sequencing of the KF8 and CX1 isolates further confirmed these two recombination events.

**Table 2:** Recombination detected with the seven methods used in the RDP4 program.

Recombination detection methods	Interspecific recombination value of *p*	Intraspecific recombination value of *p*
RDP	3.9×10^−151^	1.4×10^−55^
GENECONV	1.3×10^−112^	9.8×10^−56^
BootScan	7.8×10^−143^	3.1×10^−57^
MaxChi	7.6×10^−42^	5.3×10^−26^
Chimaera	1.7×10^−46^	2.6×10^−29^
SiScan	3.7×10^−62^	9.5×10^−40^
3Seq	2.6×10^−11^	1.3×10^−11^

### Phylogenetic Analysis

GTR+G+I substitution model, suggested by jModelTest2 (Darriba et al., 2012), was used for the phylogenetic tree construction based on nucleotide sequences. Phylogenetic analysis of sequences from nt 1–2,089 (including 5'-UTR, coding regions of P1 and part of HC-pro) revealed that isolates KF8, KF17, and CX1 were clustered with the PRSV-SK isolate from South Korea within the PRSV clade (Figure 3A), while phylogenetic analysis of sequences from 2,090 to 10,325 (including coding regions of part of HC-pro and other proteins and 3'-UTR) clustered the three isolates into the ZTMV clade (Figure 3B). These results further verified that these three isolates are indeed interspecific recombinant viruses from PRSV and ZTMV. Furthermore, phylogenetic tree constructed using sequences from nt 2,090–4,089 combined with sequences from nt 8,980–10,325 revealed that the CX1 isolate was clustered with KF8 and KF17 (Figure 3C), whereas CX1 was clustered with ZTMV-E11045 and ZTMV-XW in the phylogenetic tree constructed using sequences from nt 2,090–4,089 combined with sequences from nt 4,090–8,979 (Figure 3D). These results also confirmed that the CX1 isolate was an intraspecific recombinant virus in the ZTMV population.

**Figure 6:**
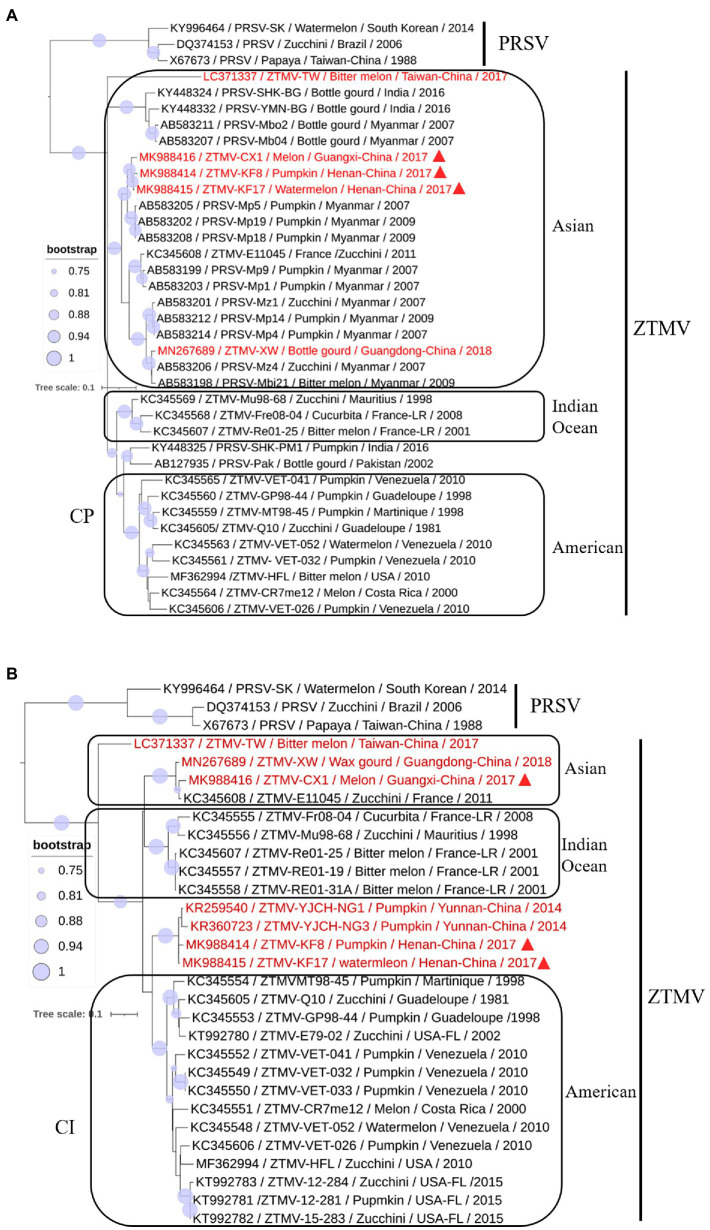
Phylogenetic analysis of coding regions of CI **(A)** and CP **(B)**. Accession number, isolate name, host, collected location and collected year are provided in nodes. Isolates with red text represent ZTMV isolates from China and those marked by red triangles represent recombination isolates identified in this study.

A phylogenetic tree (Figure 6A) constructed with the coding region of CP from 34 members (including 16 ZTMV isolates that are wrongly proposed as PRSV isolates in GenBank) of the ZTMV cluster showed that all isolates except PRSV-SHK-PM1 (accession number KY448325) and PRSV-Pak (accession number AB127935) were grouped into three subgroups: Asian, American, and the Indian ocean, related to their geographical origins (Romay et al., 2014). The Asian subgroup contained all five isolates from China (including three in this study), 12 from Myanmar, two from Indian and one from France. Furthermore, the phylogenetic tree (Figure 6B) constructed with nucleotide sequences of CI coding region from 27 members of the ZTMV cluster showed that the ZTMV cluster were grouped into three subgroups but different from the tree constructed with the nt sequences of CP. The Indian Ocean subgroup contained all isolates from Indian Ocean, and subgroup Asia contained the E11045 isolate from France and three isolates (TW, CX1, and XW) from southern of China. Subgroup America included all isolates from America. The four Chinese isolates, KF8 and KF17 from Henan province, and YJCH-NG1 (accession number KR259540) and YJCH-NG3 (accession number KR360723) from Yunnan province did not cluster with other Asian isolates but were more closely related to the subgroup America. Phylogeny based on nt sequences of Nla-pro was similar to that of CI. Phylogeny based on partial nt sequences of P3 showed that YSP-HG2 (accession number KU058173) and YSK-XHL2 (accession number KU058173), also from Yunnan of China, were grouped into a cluster with four isolates (KF8, KF17, CX1, and XW) from the mainland of China, similar to that in the CP tree (data not shown).

### Construction and Characterization of Infectious cDNA Clones

**Figure 7:**
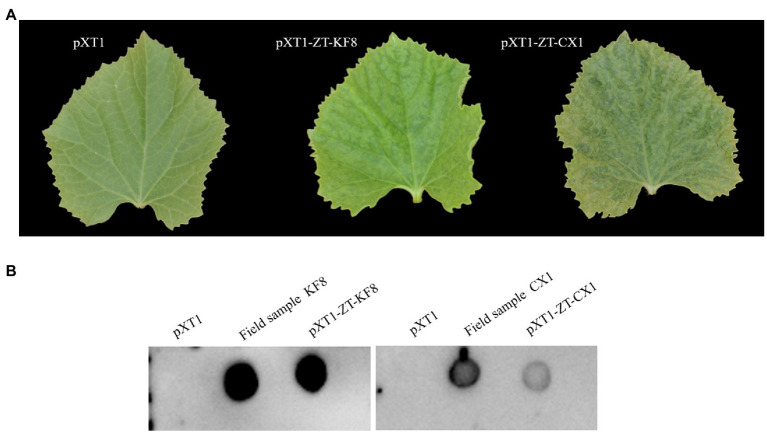
Recovery of ZTMV from infectious cDNA clones. **(A)** Symptoms shown on the non-inoculated upper leaf at 14 dpi. **(B)** Detection of viral RNA of ZTMV by dot blot hybridization.

KF17 and KF8 isolates, collected from the same field, had very high identity (97.8%) in their genome sequences (Figure 4). Therefore, we selected one of them, KF8, along with CX1, for biological characterizations. Two fragments, 6,575-bp and 3,633-bp in length, covered the complete sequence of ZTMV-KF8 (Supplementary Figure 1C), were amplified from cDNA with two primer pairs (5utrL&6700R and 6700F&3utrL, respectively) using LD-PCR. Both of them were synchronously cloned into a linearized pXT1 vector (Supplementary Figure 1C) by a homologous recombination reaction, resulting in constructing a plasmid, named pXT1-ZT-KF8, that contained a complete sequence of ZTMV-KF8. Another fragment, 10,395-bp in length (Supplementary Figure 1C), was obtained from amplifying cDNA of the ZTMV-CX1 isolate using the primer pair 5utrL&3utrL. The plasmid containing the complete sequence of ZTMV isolates CX1, named pXT1-ZT-CX1, was constructed. After 7days post-inoculation (dpi), early symptoms, as well as mild mosaic or vine clearing were observed in the new non-inoculated upper leaves of melon plants injected by agrobacterium with pXT1-ZT-KF8 and pXT1-ZT-CX1 plasmid, respectively. At 14 dpi, obvious mosaic was shown on leaves of seven out of 10 of inoculated melon plants, but no symptoms could be observed on mock-inoculated plants (Figure 7A). The presence of ZTMV-KF8 and ZTMV-CX1 isolates in the plants was confirmed by RT-PCR and dot-blot hybridization (Figure 7B), and the absence of ZYMV, WMV, PRSV, and CMV was confirmed by RT-PCR using species-specific primers. The results of DAS-ELISA also revealed that the two isolates of ZTMV were not able to react against ZYMV, WMV, PRSV, and CMV antiserum. Two ZTMV isolates were successfully recovered from infectious cDNA clones, and the typical viral disease symptoms were shown after inoculation of them on melons.

### Determination of Transmissibility and Host Ranges

After 14days of mechanical inoculation with the sap of ZTMV-KF8 and ZTMV-CX1 isolates derived from infectious cDNA clones, 13 out of 15 and 10 out of 15 melon plants showed the viral disease symptoms as well as mosaic, respectively. Similarly, after 14days of inoculation with the two isolates through aphid, all 10 of inoculated plants showed the symptoms same as ones of mechanical inoculation. The detection results of RT-PCR using ZTMV species-specific primers coincided with the presence of the symptoms.

After mechanical inoculation, systemic symptoms were observed in seven tested members of *Cucurbitaceae*. The two isolates of ZTMV produced mosaic or mottle in most *Cucurbitaceae*, chlorotic spots in orient melon, which was visible at 15 dpi and later developed into blistering in muskmelon and bottle gourd, curling in orient melon, and leaf deformations in zucchini, pumpkin and bottle gourd visible at 30 dpi (Table 1 and Supplementary Figure 2). No systemic infection was detected in papaya and five indicator hosts, including three *Solanaceae* and two *Chenopodiaceae*, in three independent repeats. The negative results detected by RT-PCR coincided with the lack of systemic symptoms.

### Prevalence of the Recombinants

RT-PCR was performed with two pairs of primers (600F&2400R and 9300F&3utr) for all samples collected from Guangxi and Henan provinces in 2years. Twelve out of 60 (20%) were found positive for ZTMV, including two melons (2017-cx01 and 2017-cx02), one pumpkin (2017-kf08), and three watermelons (2017-kf17, 2017-kf23, and 2017-kf25) out of 29 cucurbit samples (four pumpkins, six melons, and 19 watermelons) collected in 2017, and five pumpkins (2018-kf17, 2018-kf18, 2018-kf19, 2018-kf120, and 2018-kf29) and one watermelon (2018-kf04) out of 31 cucurbit samples (17 pumpkins and 14 watermelons) collected in 2018. All PCR products were sequenced using Sanger dideoxy DNA sequencing. The nucleotide sequences of PCR products using the 600F&2400R primer pair (Figure 2C and Supplemental Figure 1A), covering the interspecific recombination site, were about 1,844nt in length. The nt sequence identity between the two positive samples from Guangxi was 99.1%, and among all nine positive samples from Henan were above 98.2%. Sequences of PCR products obtained with the 9300F&3utr primer pair (Supplemental Figure 1A, bottom) were about 1,047nt in length. Likewise, the nt sequence identity between the two positive samples from Guangxi was 99.4%, and among all nine positive samples from Henan were above 98.6%. Comparison of these sequences against the GenBank nucleotide database revealed that the former product of ZTMV isolate from 2017-kf08 shared the highest nucleotide sequence identity (approximately 85%) with PRSV (KY996464), while the latter product shared the highest nucleotide sequence identity (approximately 92%) with ZTMV (KC345608).

## Discussion

Zucchini tigre mosaic potyvirus has been detected in cucurbit crops grown in Asia, Europe, and America, as well as some Caribbean and Indian Ocean islands (Romay et al., 2014; Xiao et al., 2016; Desbiez et al., 2017; Abdalla and Ali, 2018; Wang et al., 2019); however, to date only seven complete genome sequences of ZTMV are available in GenBank (Figure 3). In China, ZTMV was first reported in pumpkin, zucchini, and cucumber of Yunnan province, located in southwestern China near Myanmar. The isolates of ZTMV from Yunnan, lack of complete genome sequences, only have four sequences of partial P3 and CI coding regions in GenBank. In recent years, two complete genome sequences of ZTMV isolates, one from bottle gourd in Guangdong and the other from bitter melon in Taiwan, have been deposited in GenBank under accession numbers MN267685 and LC371337, respectively. In this study, we identified and characterized interspecific recombination isolates of ZTMV from cucurbit crops (watermelon, pumpkin, and melon) in China using next-generation sequencing (NGS) and molecular approaches. As far as we know, our study is the first to detect and document the recombination isolates of ZTMV, which originated from a recombination event located in the coding region of HC-pro between ZTMV and PRSV. ZTMV has long been overlooked in cucurbit production, because it has the closest phylogenetic relationship with PRSV, shares the same host ranges and causes similar symptoms compared to other cucurbit-infecting potyviruses, including PRSV, ZYMV, and WMV (Romay et al., 2014). Due to the acquisition of molecular characteristics and the development of discriminating diagnostic tools for virus, such as PCR and NGS, ZTMV even the recombinant ZTMV isolates can now be readily distinguished from PRSV. In this study, we found that the host ranges and induced symptoms of the recombinant ZTMV isolates (Table 1) are indeed not significantly different from those of PRSV and ZTMV. According to the 2-year investigation in the same field in Kaifeng of Henan province in China (Supplementary Table 1 and Supplementary Figure 1A), the interspecific recombination isolates have colonized cucurbits cultivated in the field and formed a stable virus entity with other cucurbit-infecting viruses, such as ZYVM, WMV, and CMV.

The three isolates are interspecific recombination viruses, with their minor parts of the genome derived from 5'-terminal region of PRSV (Figure 5A). However, we suggested that they should belong to ZTMV based on multiple alignments of complete nt and aa sequences of polyproteins (Figure 4), and according to species demarcation criteria for genus *Potyvirus* (Adams et al., 2005; Wylie et al., 2017). However, the amino acid sequence identities of CP between KF8 and ZTMV-HFL (72.4%) and between KF8 and ZTMV-XW (81.8%) were lower than those between KF8 and PRSV-SK (85.7%) and between KF8 and PRSV-X67673 (86.7%; Supplementary Table 6). Considering only the CP, according to the option species demarcation criterion of 80% amino acid identity (Adams et al., 2005), ZTMV could not be distinguished from PRSV. On the contrary, amino acid sequence identities of CI of KF8 with other 10 ZTMV isolates were 88.5–98.7, and 80.3 and 80.5% with PRSV-SK and PRSV-X67673, respectively. The option species demarcation criterion is 88% amino acid identity for CI (Adams et al., 2005). It has been discussed that ZTMV can be distinguished from PRSV using the coding region of CI more suitable than using the CP (Adams et al., 2005; Romay et al., 2014). A similar conclusion cloud be drawn from phylogenetic analysis based on CP nt sequences (Figure 6A), which indicated that some isolates collected from Asia (Myanmar, India, and Pakistan) between 2002 and 2016 that were designed as PRSV in GenBank, were clustered into the ZTMV group.

With the advance and reduced cost of NGS, it has a significant advantage in the discovery of novel viruses, identification of diverse strains and unravelling the virome of many crops compared to the conventional methods for virus detection (Villamor et al., 2019). In this study, our work was triggered by an apparent clue that suspected interspecific recombination isolates of ZTMV were present in cucurbits, suggested by the analysis of several sRNA sequencing data from field samples. The clue of the presence of interspecific recombination isolates should owe to the visual diagrams of contig mapping to the reference viral genome provided by VirusDetect (Zheng et al., 2017; Figure 1). In our previous work (Peng et al., 2019), the identification of an interspecific recombination virus, zucchini aphid-borne yellows virus belonging to genus *polerovirus*, was also triggered by the analysis of an RNA-seq data using the VirusDetect pipeline. From our experience, it is feasible that interspecific recombination events could be identified from suitable NGS data using the pipeline, according to visual diagrams and associated information (range of coverage, depth etc. shown in Supplementary Table 4) provided by the pipeline. However, intraspecific recombination isolates could not be identified with NGS data using the pipeline, such as an intraspecific recombination event among ZTMV-CX1, ZTMV-KF8 and ZTMV-E11045 (Figure 5B).

RNA recombination is common and is one of the driving forces of evolution in potyvirus populations (Bujarski, 2013). A lot of intraspecies recombination events have been reported in potyviruses, but only two interspecies recombination events have been experimentally confirmed, namely WMV and SuWMV (Gibbs et al., 2020), and their breakpoints of recombination are both in the coding region of P1 (Desbiez and Lecoq, 2004; Desbiez et al., 2017). P1 is referred to as a mysterious protein of family *Potyviridae*, since it varies in length and sequence, and is deemed to be the key factor of potyviruses to adapt to various hosts and environments (Rohožková and Navrátil, 2011). Some studies indicate that genetic changes of the N-terminal of the P1 coding region have less lethal risk of recombinant isolates than other viral proteins (Valli et al., 2007; Desbiez et al., 2017). In this study, the breakpoints of recombinant ZTMV isolates are near the N'-terminal of HC-Pro (Figure 1A), and the genetic exchange involves whole P1 and part of HC-Pro. This recombinant pattern has not been found in potyviruses involving interspecific recombination but found in an intraspecific isolate of PRSV (Maina et al., 2019). The PRSV-XM isolate (accession number KY933061), collected from papaya in China, is a recombinant with PRSV-SK (KY996464) as a major parent with a minor 5'-terminal region (coding region of P1 and part of HC-Pro) of PRSV-HN-1, an isolate from Papaya in China. Besides the interspecific recombination, we also found an intraspecific recombination (Figure 5B) among the ZTMV population. CX1 isolate is a recombinant with the KF8 isolate as a major parent with the coding region of 6K1, CI, 6K2, VPg, Nla-pro, and part of Nlb of ZTMV-XW isolate. Both KF8 collected from Henan and CX1 isolate collected from Guangxi belong to ZTMV interspecific recombinant, whereas XW isolate collected from Guangdong and CX1 have the same geographical origin, both of which are in southern China. Henan is located in central China, more than 1,500km away from Guangdong and Guangxi, and the climatic types are very different from each other. Identification of the intraspecific recombination indicated that long-distance genetic connectivity could have occurred among ZTMV populations in China.

Based on the phylogeny of CP nt sequences, the ZTMV population fell into three subgroups (namely the Asia, the America, and the Indian Ocean), related to their geographical origins (Romay et al., 2014). In this study, the same analysis based on the CP coding region (Figure 6A), was in accordance with the previous report (Romay et al., 2014), and the three interspecific isolates from China were grouped into the Asian group as expected. The E11045 isolate from France was grouped with other isolates from Asia. Romay et al. (2014) provided a possible explanation that the isolate E11045 invaded France by an accidental introduction. Another two isolates, one from India (accession number KY448345) and the other from Pakistan (accession number AB127935), were unexpectedly clustered between the India ocean subgroup and the American subgroup (Figure 6A). These results suggest that there is more genetic diversity in isolates from the South Asia subcontinent (India and Pakistan), which is consistent with the finding from a previous study (Castillo et al., 2011) that PRSV from India have the highest diversity of CP nt sequence. On the other hand, phylogenetic analysis on CI (Figure 6B) showed that the ZTMV of KF8, KF17 and other Yunnan isolates have a closer relationship with American isolates than with other Asian isolates. Similarly, an uncertain recombinant event (data not shown) was identified in the coding region of CI, 6K2, Vpg, and Nla-pro among KF8 isolate, CX1 isolate and VET-026 isolate (accession number KC345606) from Venezuela. These results implied that there could be genetic connections between Asian isolates and American isolates.

In this study, we identified and characterized interspecific recombinant viruses between zucchini tigre mosaic virus and Papaya ringspot virus in cucurbits. Despite the origin from interspecific recombination, we proposed that these viruses still belong to zucchini tigre mosaic virus according to their genome characteristics and biological features. We infer that the recombinant virus is epidemic in cucurbits with lower prevalence than other viruses (ZYMV, WMV etc.). In the future, we should develop efficient methods to monitor the incidence and damage of the recombinant ZTMV in cucurbits countrywide. On the other hand, our study provided new insights into the origin and evolution of ZTMV and PRSV.

## Data Availability Statement

The datasets presented in this study can be found in online repositories. The names of the repository/repositories and accession number(s) can be found in the article/Supplementary Material.

## Author Contributions

BP and QG contributed to conceptualization and writing. QG contributed to funding acquisition and supervision. BP contributed to investigation, sampling, and analysis of data. BP, LL, BK, and HW performed the experiment. ZF helped to perform the analysis of sRNA sequencing data and writing. All authors contributed to the article and approved the submitted version.

## Funding

Financial support was provided by the China Agriculture Research System of MOF and MARA (CARS-25), and Central Public-interest Scientific Institution Basal Research Fund (No. 1610192021401).

## Conflict of Interest

The authors declare that the research was conducted in the absence of any commercial or financial relationships that could be construed as a potential conflict of interest.

The reviewer XW declared a shared affiliation with some of the authors, BP, LL, HW, BK, and QG to the handling editor at time of review.

## Publisher’s Note

All claims expressed in this article are solely those of the authors and do not necessarily represent those of their affiliated organizations, or those of the publisher, the editors and the reviewers. Any product that may be evaluated in this article, or claim that may be made by its manufacturer, is not guaranteed or endorsed by the publisher.

## Footnotes

^1^ http://www.lynnon.com/

^2^ https://www.ncbi.nlm.nih.gov/orffinder/

^3^ https://itol.embl.de/

## References

[ref1] Abdalla O. A. Ali A. (2018). Molecular characterization reveals that squash chlorosis mottling virus and zucchini tigré mosaic virus are the same newly emerging potyvirus. Arch. Virol. 163, 777–780. doi: 10.1007/s00705-017-3657-x, PMID: 29164402

[ref2] Adams M. Antoniw J. Fauquet C. (2005). Molecular criteria for genus and species discrimination within the family Potyviridae. Arch. Virol. 150, 459–479. doi: 10.1007/s00705-004-0440-6, PMID: 15592889

[ref3] Bujarski J. J. (2013). Genetic recombination in plant-infecting messenger-sense RNA viruses: overview and research perspectives. Front. Plant Sci. 4:68. doi: 10.3389/fpls.2013.00068, PMID: 23533000 PMC3607795

[ref4] Castillo X. O. Fermin G. Tabima J. Rojas Y. Tennant P. Fuchs M.. (2011). Phylogeography and molecular epidemiology of papaya ringspot virus. Virus Res. 159, 132–140. doi: 10.1016/j.virusres.2011.04.011, PMID: 21549774

[ref5] Darriba D. Taboada G. L. Doallo R. Posada D. (2012). jModelTest 2: more models, new heuristics and parallel computing. Nat. Methods 9:772. doi: 10.1038/nmeth.2109, PMID: 22847109 PMC4594756

[ref6] Desbiez C. Lecoq H. (2004). The nucleotide sequence of watermelon mosaic virus (WMV, Potyvirus) reveals interspecific recombination between two related potyviruses in the 5′ part of the genome. Arch. Virol. 149, 1619–1632. doi: 10.1007/s00705-004-0340-9, PMID: 15290385

[ref7] Desbiez C. Wipf-Scheibel C. Millot P. Verdin E. Dafalla G. Lecoq H. (2017). New species in the papaya ringspot virus cluster: insights into the evolution of the PRSV lineage. Virus Res. 241, 88–94. doi: 10.1016/j.virusres.2017.06.022, PMID: 28669763

[ref8] Garcia-Mas J. Benjak A. Sanseverino W. Bourgeois M. Mir G. González V. M.. (2012). The genome of melon (*Cucumis melo* L.). Proc. Natl. Acad. Sci. U. S. A. 109, 11872–11877. doi: 10.1073/pnas.1205415109, PMID: 22753475 PMC3406823

[ref9] Gibbs A. J. Hajizadeh M. Ohshima K. Jones R. A. (2020). The potyviruses: an evolutionary synthesis is emerging. Viruses 12:132. doi: 10.3390/v12020132, PMID: 31979056 PMC7077269

[ref10] Guo S. Zhang J. Sun H. Salse J. Lucas W. J. Zhang H.. (2013). The draft genome of watermelon (*Citrullus lanatus*) and resequencing of 20 diverse accessions. Nat. Genet. 45:51. doi: 10.1038/ng.2470, PMID: 23179023

[ref11] Ibaba J. Laing M. Gubba A. (2016). Zucchini shoestring virus: a distinct potyvirus in the papaya ringspot virus cluster. Arch. Virol. 161, 2321–2323. doi: 10.1007/s00705-016-2899-3, PMID: 27216927

[ref12] King A. M. Q. Adams M. J. Carstens E. B. Lefkowitz E. J. (2011). Virus Taxonomy: Ninth Report of the International Committee on Taxonomy of Viruse. Amsterdam: Elsevier Academic.

[ref13] Kumar S. Stecher G. Li M. Knyaz C. Tamura K. (2018). MEGA X: molecular evolutionary genetics analysis across computing platforms. Mol. Biol. Evol. 35, 1547–1549. doi: 10.1093/molbev/msy096, PMID: 29722887 PMC5967553

[ref14] Lecoq H. Desbiez C. (2012). “Viruses of cucurbit crops in the Mediterranean region: an ever-changing picture,” in Advances in Virus Research. eds. Maramorosch K. Shatkin A. Murphy F. A. (San Diego, CA, USA: Elsevier), 67–126. 10.1016/B978-0-12-394314-9.00003-8 22682166

[ref15] Liu Y. Li F. Zhang S. Gao X. Xie Y. Zhang A.. (2019b). Identification, distribution and occurrence of viruses in the main vegetables of China. Sci. Agric. Sin. 52, 239–261.

[ref16] Liu L. Peng B. Zhang Z. Wu Y. Miras M. Aranda M. A.. (2017). Exploring different mutations at a single amino acid position of cucumber green mottle mosaic virus replicase to attain stable symptom attenuation. Phytopathology 107, 1080–1086. doi: 10.1094/PHYTO-03-17-0107-R, PMID: 28545349

[ref17] Liu L. Xie K. Tsekpuia A. R. Peng B. Liu M. Gu Q. (2019a). Construction and biological characterization of an *Agrobacterium*-mediated infectious cDNA of squash mosaic virus. Virus Res. 274:197766. doi: 10.1016/j.virusres.2019.197766, PMID: 31560966

[ref18] Maina S. Barbetti M. J. Edwards O. R. Minemba D. Areke M. W. Jones R. A. (2019). Genetic connectivity between papaya ringspot virus genomes from Papua New Guinea and Northern Australia, and new recombination insights. Plant Dis. 103, 737–747. doi: 10.1094/PDIS-07-18-1136-RE, PMID: 30856073

[ref19] Martin D. P. Murrell B. Golden M. Khoosal A. Muhire B. (2015). RDP4: detection and analysis of recombination patterns in virus genomes. Virus Evol. 1:1. doi: 10.1093/ve/vev003, PMID: 27774277 PMC5014473

[ref20] Moury B. Desbiez C. (2020). Host range evolution of potyviruses: a global phylogenetic analysis. Viruses 12:111. doi: 10.3390/v12010111, PMID: 31963241 PMC7020010

[ref21] Pasin F. Simón-Mateo C. García J. A. (2014). The hypervariable amino-terminus of P1 protease modulates potyviral replication and host defense responses. PLoS Pathog. 10:e1003985. doi: 10.1371/journal.ppat.1003985, PMID: 24603811 PMC3946448

[ref22] Peng B. Kang B. Wu H. Liu L. Liu L. Fei Z.. (2019). Detection and genome characterization of a novel member of the genus Polerovirus from zucchini (*Cucurbita pepo*) in China. Arch. Virol. 164, 2187–2191. doi: 10.1007/s00705-019-04217-w, PMID: 31123960

[ref23] Perotto M. C. Pozzi E. A. Celli M. G. Luciani C. E. Mitidieri M. S. Conci V. C. (2018). Identification and characterization of a new potyvirus infecting cucurbits. Arch. Virol. 163, 719–724. doi: 10.1007/s00705-017-3660-2, PMID: 29196817

[ref24] Quiot-Douine L. Purcifull D. Hiebert E. De Mejia M. (1986). Serological relationships and in vitro translation of an antigenically distinct strain of papaya ringspot virus. Phytopathology 76, 346–351. doi: 10.1094/Phyto-76-346

[ref25] Rohožková J. Navrátil M. (2011). P1 peptidase–a mysterious protein of family Potyviridae. J. Biosci. 36, 189–200. doi: 10.1007/s12038-011-9020-6, PMID: 21451259

[ref26] Romay G. Lecoq H. Desbiez C. (2014). Zucchini tigré mosaic virus is a distinct potyvirus in the papaya ringspot virus cluster: molecular and biological insights. Arch. Virol. 159, 277–289. doi: 10.1007/s00705-013-1798-0, PMID: 23979176

[ref27] Salvador B. Saenz P. YangÜez E. Quiot J. B. Quiot L. Delgadillo M. O.. (2008). Host-specific effect of P1 exchange between two potyviruses. Mol. Plant Pathol. 9, 147–155. doi: 10.1111/j.1364-3703.2007.00450.x, PMID: 18705848 PMC6640519

[ref28] Sievers F. Higgins D. G. (2014). Clustal omega. Curr. Protoc. Bioinformatics 48, 3.13.11–3.13.16. doi: 10.1002/0471250953.bi0313s48, PMID: 25501942

[ref29] Sun H. Wu S. Zhang G. Jiao C. Guo S. Ren Y.. (2017). Karyotype stability and unbiased fractionation in the paleo-allotetraploid Cucurbita genomes. Mol. Plant 10, 1293–1306. doi: 10.1016/j.molp.2017.09.003, PMID: 28917590

[ref30] Valli A. Lopez-Moya J. J. Garcia J. A. (2007). Recombination and gene duplication in the evolutionary diversification of P1 proteins in the family Potyviridae. J. Gen. Virol. 88, 1016–1028. doi: 10.1099/vir.0.82402-0, PMID: 17325376

[ref31] Villamor D. Ho T. Al Rwahnih M. Martin R. Tzanetakis I. (2019). High throughput sequencing for plant virus detection and discovery. Phytopathology 109, 716–725. doi: 10.1094/PHYTO-07-18-0257-RVW, PMID: 30801236

[ref32] Wang D. Boluk G. Quinto E. A. Hamim I. Borth W. B. Melzer M.. (2019). First report of zucchini tigre mosaic virus infecting bitter melon (*Momordica charantia*) in Hawaii. Plant Dis. 103:1047. doi: 10.1094/PDIS-08-18-1391-PDN

[ref33] Wylie S. J. Adams M. Chalam C. Kreuze J. López-Moya J. J. Ohshima K.. (2017). ICTV virus taxonomy profile: Potyviridae. J. Gen. Virol. 98:352. doi: 10.1099/jgv.0.000960, PMID: 28366187 PMC5797945

[ref34] Xiao L. Li Y. Tan G. Lan P. Zhong L. Liu Y.. (2016). First report of zucchini tigre mosaic virus infecting several cucurbit plants in China. Plant Dis. 100:1253. doi: 10.1094/PDIS-11-15-1318-PDN

[ref35] Yakoubi S. Lecoq H. Desbiez C. (2008). Algerian watermelon mosaic virus (AWMV): a new potyvirus species in the PRSV cluster. Virus Genes 37, 103–109. doi: 10.1007/s11262-008-0237-x, PMID: 18484176

[ref36] Yao M. Zhang T. Tian Z. Wang Y. Tao X. (2011). Construction of *Agrobacterium*-mediated cucumber mosaic virus infectious cDNA clones and 2b deletion viral vector. Sci. Agric. Sin. 44, 3060–3068.

[ref37] Zerbino D. R. Birney E. (2008). Velvet: algorithms for de novo short read assembly using de Bruijn graphs. Genome Res. 18, 821–829. doi: 10.1101/gr.074492.107, PMID: 18349386 PMC2336801

[ref38] Zheng Y. Gao S. Padmanabhan C. Li R. Galvez M. Gutierrez D.. (2017). VirusDetect: an automated pipeline for efficient virus discovery using deep sequencing of small RNAs. Virology 500, 130–138. doi: 10.1016/j.virol.2016.10.017, PMID: 27825033

