# Supplementary material for: Interspecific Recombination Between Zucchini Tigre Mosaic Virus and Papaya Ringspot Virus Infecting Cucurbits in China
Source: Front Microbiol. 2021 Nov 3;12:773992. doi: 10.3389/fmicb.2021.773992 (PMC8595935; doi:10.3389/fmicb.2021.773992)
Supplement: Supplementary file 1 [file Data_Sheet_1.docx]

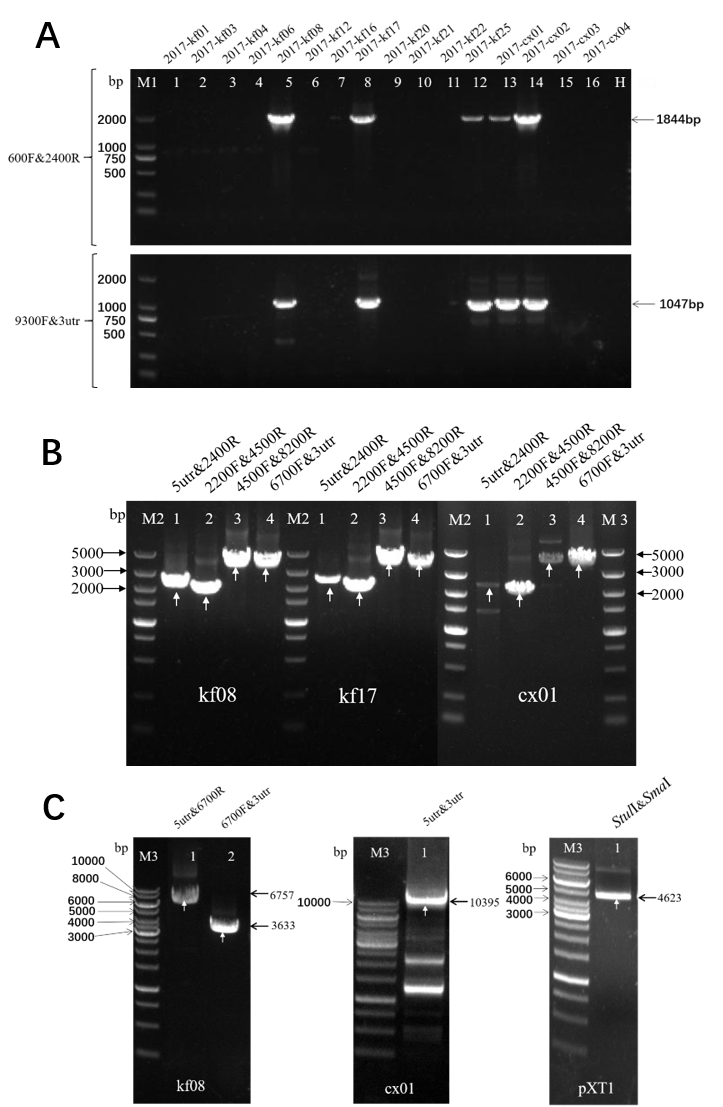


Supplementary Fig. 1 Electrophoresis of amplified fragments of the ZTMV genome and digestion of the pXT1 vector. (A) Verification of recombination of ZTMV by RT-PCR. (B) Fragments covering the complete genomes of three ZTMV isolates amplified with four independent RT-PCR. (C) Fragments for the construction of infectious cDNA clones amplified by long-distance RT-PCR and digestion of the pXT1 vector by *stul*1 and *sma*1 double restriction enzymes. M1, Takara DL2000 marker. M2, Takara DL5000 marker. M3, Takara 1kb ladder marker.


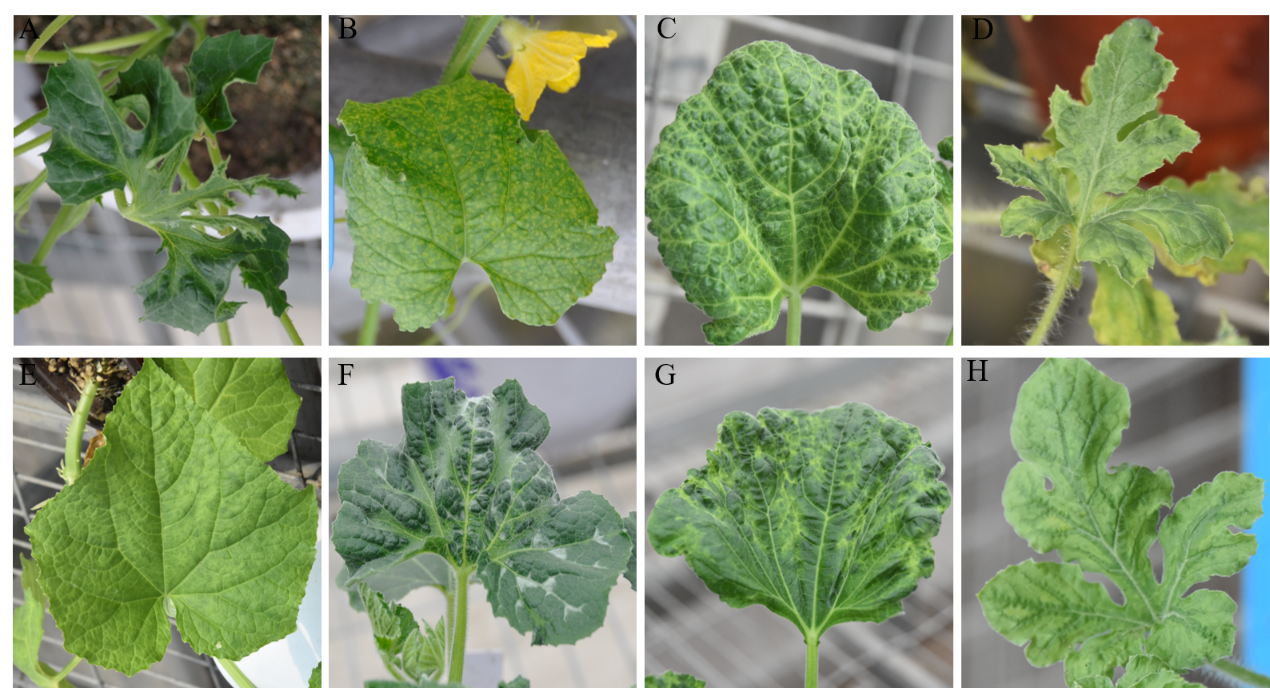


Supplementary Fig. 2 symptoms shown on cucurbits at 30 dpi. (A-D), symptoms on zucchini (A), oriental melon (B), bottle gourd (C) and watermelon (D) inoculated with the ZTMV-CX1 isolate. (E-H), symptoms on cucumber (E), pumpkin (F) and watermelon (G, H) inoculated with ZTMV-KF8 isolate.

Supplementary Table 1: Information of samples collected from Guangxi and Henan of China

| Number | Year collection-sample name | Location | Host | Symptoms |
| --- | --- | --- | --- | --- |
| 1 | **2017-cx01** | Cenxi, Guangxi | Melon | vine yellow |
| 2 | **2017-cx02** | Cenxi, Guangxi | Melon | vine yellow |
| 3 | 2017-cx03 | Cenxi, Guangxi | Melon | mosaic |
| 4 | 2017-cx04 | Cenxi, Guangxi | Melon | mosaic |
| 5 | 2017-kf01 | Kaifeng, Henan | Pumpkin | mosaic |
| 6 | 2017-kf02 | Kaifeng, Henan | Pumpkin | mosaic |
| 7 | 2017-kf03 | Kaifeng, Henan | watermelon | mosaic, crinkle |
| 8 | 2017-kf04 | Kaifeng, Henan | watermelon | mosaic |
| 9 | 2017-kf05 | Kaifeng, Henan | watermelon | yellow, mosaic |
| 10 | 2017-kf06 | Kaifeng, Henan | watermelon | mosaic |
| 11 | 2017-kf07 | Kaifeng, Henan | watermelon | yellow |
| 12 | **2017-kf08** | Kaifeng, Henan | Pumpkin | yellow, ringspot |
| 13 | 2017-kf09 | Kaifeng, Henan | watermelon | mosaic |
| 14 | 2017-kf10 | Kaifeng, Henan | Melon | mosaic |
| 15 | 2017-kf11 | Kaifeng, Henan | Melon | yellow，mosaic |
| 16 | 2017-kf12 | Kaifeng, Henan | watermelon | mosaic |
| 17 | 2017-kf13 | Kaifeng, Henan | watermelon | mottle |
| 18 | 2017-kf14 | Kaifeng, Henan | Pumpkin | mosaic |
| 19 | 2017-kf15 | Kaifeng, Henan | watermelon | yellow |
| 20 | 2017-kf16 | Kaifeng, Henan | watermelon | mosaic |
| 21 | **2017-kf17** | Kaifeng, Henan | watermelon | mosaic |
| 22 | 2017-kf18 | Kaifeng, Henan | watermelon | mosaic |
| 23 | 2017-kf19 | Kaifeng, Henan | watermelon | mosaic |
| 24 | 2017-kf20 | Kaifeng, Henan | watermelon | mosaic, crinkle |
| 25 | 2017-kf21 | Kaifeng, Henan | watermelon | mosaic |
| 26 | 2017-kf22 | Kaifeng, Henan | watermelon | mosaic |
| 27 | **2017-kf23** | Kaifeng, Henan | watermelon | mosaic |
| 28 | 2017-kf24 | Kaifeng, Henan | watermelon | mosaic |
| 29 | **2017-kf25** | Kaifeng, Henan | watermelon | mosaic, yellow |
| 30 | 2018-kf01 | Kaifeng, Henan | Watermelon | mosaic |
| 31 | 2018-kf02 | Kaifeng, Henan | Watermelon | mosaic |
| 32 | 2018-kf03 | Kaifeng, Henan | Watermelon | mosaic |
| 33 | **2018**-**kf04** | Kaifeng, Henan | Watermelon | mosaic |
| 34 | 2018-kf05 | Kaifeng, Henan | Watermelon | mosaic |
| 35 | 2018-kf06 | Kaifeng, Henan | Pumpkin | yellow, mosaic |
| 36 | 2018-kf07 | Kaifeng, Henan | Watermelon | mosaic |
| 37 | 2018-kf08 | Kaifeng, Henan | Pumpkin | mosaic |
| 38 | 2018-kf09 | Kaifeng, Henan | Pumpkin | mosaic, yellow |
| 39 | 2018-kf10 | Kaifeng, Henan | Pumpkin | mosaic |
| 40 | 2018-kf11 | Kaifeng, Henan | Watermelon | mosaic |
| 41 | 2018-kf12 | Kaifeng, Henan | Pumpkin | mosaic |
| 42 | 2018-kf13 | Kaifeng, Henan | Pumpkin | mosaic,yellow |
| 43 | 2018-kf14 | Kaifeng, Henan | Watermelon | mosaic |
| 44 | 2018-kf15 | Kaifeng, Henan | Watermelon | mosaic |
| 45 | 2018-kf16 | Kaifeng, Henan | Pumpkin | mosaic |
| 46 | **2018-kf17** | Kaifeng, Henan | Pumpkin | mosaic |
| 47 | **2018-kf18** | Kaifeng, Henan | Pumpkin | mosaic |
| 48 | **2018-kf19** | Kaifeng, Henan | Pumpkin | mosaic |
| 49 | **2018-kf20** | Kaifeng, Henan | Pumpkin | mosaic |
| 50 | 2018-kf21 | Kaifeng, Henan | Pumpkin | mosaic |
| 51 | 2018-kf22 | Kaifeng, Henan | Watermelon | mosaic |
| 52 | 2018-kf23 | Kaifeng, Henan | Watermelon | mosaic |
| 53 | 2018-kf24 | Kaifeng, Henan | Watermelon | mosaic |
| 54 | 2018-kf25 | Kaifeng, Henan | Pumpkin | mosaic, yellow |
| 55 | 2018-kf26 | Kaifeng, Henan | Pumpkin | mosaic |
| 56 | 2018-kf27 | Kaifeng, Henan | Pumpkin | mosaic |
| 57 | 2018-kf28 | Kaifeng, Henan | Pumpkin | mosaic |
| 58 | **2018-kf29** | Kaifeng, Henan | Pumpkin | mosaic |
| 59 | 2018-kf30 | Kaifeng, Henan | Watermelon | mosaic |
| 60 | 2018-kf31 | Kaifeng, Henan | Watermelon | mosaic |

Samples marked bold are positive for ZTMV.

Supplementary Table 2: Information of selected samples for mixture of small RNA sequencing

| Sample No. | Viruses identified by small RNA-seq（coverage） | Host | Symptom | Small RNA-seq No | Reads count |
| --- | --- | --- | --- | --- | --- |
| 2017-kf01 | ZYMV (100%), WMV (97.1%), **ZTMV (78.6%), PRSV (11.9%)** | Pumpkin | mosaic | SR1 | 23160143 |
| **2017-kf08** |  | Pumpkin | mosaic， ringspot | SR1 |  |
| 2017-kf03 | ZYMV (100%), WMV (99.2%), CiLCV (99.5%) | Watermelon | mosaic, crinkle | SR2 | 20419717 |
| 2017-kf04 |  | Watermelon | mosaic | SR2 |  |
| 2017-kf21 | ZYMV (100%), WMV (99.4%), CiLCV (97.8%) | Watermelon | mosaic | SR3 | 20128412 |
| 2017-kf22 |  | Watermelon | mosaic | SR3 |  |
| 2017-kf06 | ZYMV (100%), WMV (99.3%), | Watermelon | mosaic | SR4 | 14874695 |
| 2017-kf12 |  | Watermelon | mosaic | SR4 |  |
| 2017-kf16 |  | Watermelon | mosaic | SR4 |  |
| **2017-kf17** | WMV (99.1%), MABYV (98.7%), **ZTMV (68.3%)**, **PRSV (16.7%)**, ZYMV (97.1%) | Watermelon | mosaic, | SR5 | 15817388 |
| 2017-kf20 |  | Watermelon | mosaic, crinkle, | SR5 |  |
| **2017-kf25** |  | Watermelon | mosaic, yellow | SR5 |  |
| **2017-cx01** | SLCCV (98.4), **ZTMV (79.1)**, PRSV **(20.6%),** MYSV (98.2%), CMEV (95.4) | Melon | vine yellowing | SR6 | 17268238 |
| **2017-cx02** |  | Melon | vine yellowing | SR6 |  |
| 2017-cx03 | SLCCV (99.2%), MYSV, (98.2%), CMEV (98.4%) | Melon | Mosaic | SR7 | 15201366 |
| 2017-cx04 |  | Melon | Mosaic | SR7 |  |

Supplementary Table 3: Primers used for confirming the genome and the construction of infectious cDNA clones of ZTMV-KF8

| Primer name | Location | Sequences （5' - 3'） | Usage |
| --- | --- | --- | --- |
| ZTMV-600R | 626-601 | CCACAGTTTCAGGTTCCACAGGGTCC | 5'RACE |
| ZTMV-800R | 798-772 | GACTTCAAATCGCAAGCACTTCCAGAG | 5'RACE |
| ZTMV-600F | 597-615 | TTTCGGACCCTGTGGAACC | RT |
| ZTMV-2200F | 2231-2250 | TCTATCAAGGCTGGGAGTG | Rec,RT |
| ZTMV-2400R | 2439-2416 | CGGATGCTGTAGTGCTCGTAAC | RT |
| ZTMV-2100R | 2255-2237 | CCAGCCTTGATAGATTCAGT | Rec,RT |
| ZTMV-4500F | 4331-4352 | CTCACGGT TGACTTTGAC TTGT | Rec,RT |
| ZTMV-4500R | 4352-4333 | ACAAGTCAAAGTCAACCGTG | Rec, RT |
| ZTMV-4600F | 4491-4518 | CTACTGTCGCTTCTGAAATCGCACATAG | RT |
| ZTMV-6400R | 6302-6281 | CACCCAAAGTGAATCCGCAAAC | RT |
| ZTMV-6700F | 6716-6741 | ATCATCAGCAATGGAGGAATCAAGGC | Rec,RT |
| ZTMV-6700R | 6740-6716 | CCTTGATTCC TCCATTGCTG ATGAT | Rec,RT |
| ZTMV-8200R | 8240-8215 | CCTAATAATGTGTCAATGGGAGCGGC | RT |
| ZTMV-8300F | 8363-8384 | GGTTGGGTCTACTGTGACG | RT |
| ZTMV-9300F | 9270-9291 | GGTGCTGTGGATGCTGGAGTTAA | RT |
| ZTMV-3utr | 10301-10325 | CTCTCATTCTAGAAGATTTGAACAC | RT |
| ZTMV-5utrL | 1-40 | GTTCATTTCATTTGGAGAGGAAATAAAACATCTCAACACAACACAATCGA AAGCA | Rec |
| ZTMV-3utrL | 10325-10298 | GTGGAGATGCCATGCCGACCCTTTTTTTTTTTTTTTTTTTTTTTTTTTTTCTCTCATTCTA GAAGATTTGA ACACAC | Rec |

RACE, rapid Amplification of cDNA Ends；RT, convention RT-PCR；Rec, Recombinant fusion; Bases marked with underline are reversely complimented with terminals of the pXT1 vector.

Supplementary Table 4: Information of identified virus contigs from SR1, SR5 and SR6 aligned to ZTMV-E11045 (accession number KC345608) and PRSV-SK (accession number KY996464) genomes.

| sRNA sample id | SR1 | | SR5 | | SR6 | |
| --- | --- | --- | --- | --- | --- | --- |
| reference virus | ZTMV | PRSV | ZTMV | PRSV | ZTMV | PRSV |
| number of contigs | 26 | 7 | 26 | 10 | 27 | 13 |
| length of contigs (nt) | 42-3613 | 115-363 | 42-1785 | 45-758 | 50-5070 | 45-748 |
| coverage (%) | 78.6 | 11.9 | 68.3 | 16.7 | 79.1 | 17.6 |
| Range of hit in genome (nt) | 1-44, 2076-10340 | 92-2027 | 2112-10321 | 15-2411 | 2065-10330 | 9-2057 |
| range of identities (%) | 83-95 | 79-93 | 83-97 | 84-95 | 83-100 | 82-95 |
| average identity (%) | 87.7 | 85.4 | 88.7 | 87.1 | 92.2 | 88.5 |
| normal depth | 242.8 | 267.8 | 218.7 | 301.5 | 185.4 | 150.6 |

Supplementary Table.5 Characteristics of ZTMV genomes

| Virus-isolate | Accession number | length of genome (nt) | Length of 5'-UTR (nt) | Length of polyprotein (aa) | Length of 3'-UTR (nt) |
| --- | --- | --- | --- | --- | --- |
| ZTMV-KF8 | MK988414 | 10325 | 84 | 3350 | 187 |
| ZTMV-KF17 | MK988415 | 10328 | 85 | 3351 | 187 |
| ZTMV-CX1 | MK988416 | 10331 | 85 | 3352 | 187 |
| ZTMV-Q10 | KC345605 | 10340 | 84 | 3347 | 212 |
| ZTMV-Vet-026 | KC345606 | 10319 | 84 | 3347 | 192 |
| ZTMV-Re01-25 | KC345607 | 10346 | 112 | 3346 | 194 |
| ZTMV-E11045 | KC345608 | 10340 | 84 | 3349 | 207 |
| ZTMV-HFL | MF362994 | 10292 | 82 | 3365 | 112 |
| ZTMV-XW | MN267689 | 10320 | 84 | 3349 | 186 |
| ZTMV-TW | LC371337 | 10324 | 110 | 3341 | 188 |
| PRSV-SK | KY996464 | 10324 | 86 | 3343 | 206 |
| PRSV | X67673 | 10326 | 85 | 3344 | 226 |

Supplementary Table.6 Percent nucleotide and amino acid sequence identities between ZTMV-KF8 (Accession number MK988414) and other ZTMV and two PRSV isolates

| Accession  number | Virus isolates | Nucleotide acid | | | Amino acid | | | | | | | | | | |
| --- | --- | --- | --- | --- | --- | --- | --- | --- | --- | --- | --- | --- | --- | --- | --- |
|  |  | Genome | 5’-UTR | 3’-UTR | Polyprotein | P1 | HC-Pro | P3 | 6K1 | CI | 6K2 | Nla-Vpg | Nla-Pro | Nlb | CP |
| MK988415 | ZTMV_KF17 | 97.8 | 97.6 | 97.1 | 98.3 | 94.7 | 99.1 | 100 | 98.1 | 98.7 | 100 | 98.5 | 98.3 | 99.3 | 99 |
| MK988416 | ZTMV_CX1 | 91.4 | 97.6 | 99 | 97 | 92.3 | 98.9 | 100 | 98.1 | 96.4 | 96.5 | 96 | 97.1 | 98.3 | 98.6 |
| KC345608 | ZTMV_E11045 | 80.4 | 65.9 | 93.3 | 86.9 | 38.6 | 95.8 | 93.6 | 96.2 | 96.7 | 96.5 | 96 | 96.6 | 97.8 | 96.2 |
| MN267689 | ZTMV_XW | 81.3 | 64.6 | 94.2 | 87.1 | 39.2 | 95.8 | 96.8 | 96.2 | 96.5 | 93 | 96 | 96.6 | 97.2 | 94.8 |
| KC345605 | ZTMV_Q10 | 79.7 | 64.6 | 92.3 | 86.2 | 38.6 | 95.8 | 95.1 | 96.2 | 96.1 | 98.2 | 97 | 95 | 96.1 | 92 |
| KC345606 | ZTMV_VET-026 | 79 | 63.4 | 90.4 | 85.5 | 39.4 | 94.7 | 92.2 | 96.2 | 96.4 | 94.7 | 95 | 95.4 | 95.3 | 88.5 |
| KC345607 | ZTMV_Re01-25 | 79.6 | 57.3 | 92.3 | 86.1 | 39.6 | 95.8 | 94.2 | 92.3 | 96.1 | 96.5 | 92.1 | 94.5 | 96.5 | 94.8 |
| MF362994 | ZTMV_HFL | 78.7 | 57.3 | 91.3 | 79.2 | 37.3 | 95.4 | 91.6 | 94.2 | 88.5 | 91.2 | 75.2 | 89.1 | 86.6 | 72.4 |
| LC371337 | ZTMV_XW | 76.1 | 52.4 | 79.8 | 84.3 | 40.3 | 95 | 90.1 | 96.2 | 93.2 | 86 | 92.1 | 95.4 | 96.1 | 81.8 |
| KY996464 | PRSV_SK | 73.6 | **86.6** | 67.3 | 80.6 | **83.4** | 91.9 | 64.1 | 76.9 | 80.3 | 63.2 | 74.8 | 75.6 | 81.8 | 85.7 |
| X67673 | PRSV | 71 | 67.1 | 65.4 | 77.4 | 64.4 | 89.5 | 65.8 | 76.8 | 80.5 | 61.4 | 75.2 | 75.6 | 81.4 | 86.7 |
